# Morphological and biochemical features of *Borrelia burgdorferi* pleomorphic forms

**DOI:** 10.1099/mic.0.000027

**Published:** 2015-03

**Authors:** Leena Meriläinen, Anni Herranen, Armin Schwarzbach, Leona Gilbert

**Affiliations:** 1 Department of Biological and Environmental Sciences and NanoScience Center, University of Jyväskylä, Jyväskylä, Finland; 2 Borreliose Centrum Augsburg, Augsburg, Germany

## Abstract

The spirochaete bacterium *Borrelia burgdorferi sensu lato* is the causative agent of Lyme disease, the most common tick-borne infection in the northern hemisphere. There is a long-standing debate regarding the role of pleomorphic forms in Lyme disease pathogenesis, while very little is known about the characteristics of these morphological variants. Here, we present a comprehensive analysis of *B. burgdorferi* pleomorphic formation in different culturing conditions at physiological temperature. Interestingly, human serum induced the bacterium to change its morphology to round bodies (RBs). In addition, biofilm-like colonies in suspension were found to be part of *B. burgdorferi*’s normal *in vitro* growth. Further studies provided evidence that spherical RBs had an intact and flexible cell envelope, demonstrating that they are not cell wall deficient, or degenerative as previously implied. However, the RBs displayed lower metabolic activity compared with spirochaetes. Furthermore, our results indicated that the different pleomorphic variants were distinguishable by having unique biochemical signatures. Consequently, pleomorphic *B. burgdorferi* should be taken into consideration as being clinically relevant and influence the development of novel diagnostics and treatment protocols.

## Introduction

Lyme disease is the most commonly reported tick-borne infection in Europe and North America, and is also endemic in many areas in Asia (Mead, 2011; Radolf *et al.*, 2012). The disease is caused by the different genospecies of the spirochaete bacterium *Borrelia burgdorferi sensu lato* group (Radolf *et al.*, 2012). The cell envelope of *B. burgdorferi* consists of a protoplasmic cylinder covered by two lipid membranes (Barbour & Hayes, 1986). Between the outer and inner membrane is the periplasmic space that comprises the peptidoglycan layer and flagellar filaments (Kudryashev *et al.*, 2009). The general structure of *B. burgdorferi*’s cell envelope is exceptional and differs significantly from the typical Gram-negative bacteria. LPSs are usually outer membrane components of Gram-negative bacteria; however, *B. burgdorferi* lacks LPS (Takayama *et al.*, 1987), and has immunoreactive glycolipids instead (Ben-Menachem *et al.*, 2003). Flagella are located in the periplasmic space, while other bacteria commonly have them outside the cell (Harman *et al.*, 2013). Furthermore, the flagella not only provide the motility function, but also confine the cell shape in *B. burgdorferi* (Motaleb *et al.*, 2000).

*B. burgdorferi sensu lato* is pleomorphic, being able to change its morphology as a response to environmental conditions. The existence of pleomorphism among many bacterial species *in vitro* has been known for over a century (Mattman, 2001; Winkler, 1899). At the beginning of the 19th century, researchers proposed that spirochaete species had multiple morphologies (Berndtson, 2013). Today it is well known that many Gram-negative and Gram-positive bacteria can spontaneously or by stimulation change their morphology both *in vitro* and *in vivo* (Domingue & Woody, 1997).

Pleomorphism is commonly induced *in vitro* using compounds that either lyse the cell wall (lytic enzymes), or interfere with the cell wall synthesis, such as antibiotics (Briers *et al.*, 2012). This treatment usually leads to a complete or partial loss of peptidoglycan cell wall and the resulting cells have been called cell wall deficient (CWD), L-forms or spheroplasts (Glover *et al.*, 2009; Ranjit & Young, 2013). In addition to CWD forms, various bacteria can aggregate into biofilms (Flemming & Wingender, 2010). Furthermore, filamentous bacteria shapes of many clinically important bacteria, such as *Escherichia coli*, have been reported (Justice *et al.*, 2004). In addition to the typical spirochaete, *B. burgdorferi* is seen also as small spherical shapes (Al-Robaiy *et al.*, 2010; Alban *et al.*, 2000; Dunham-Ems *et al.*, 2012; Miklossy *et al.*, 2008), blebs (Kersten *et al.*, 1995), detaching granules or pearls (Aberer & Duray, 1991; Barbour & Hayes, 1986; Garon *et al.*, 1989), and agglomerations of spirochaetes into biofilm-like (BFL) colonies (Sapi *et al.*, 2012; Srivastava & de Silva, 2009).

Previously, the round bodies (RBs) of *B. burgdorferi* have been ambiguously named in various ways. These terms include CWD and L-forms, spheroplasts, protoplasts, propagules and even cysts (Domingue & Woody, 1997; Stricker & Johnson, 2011). Nonetheless, all of these labels describe the same spherical structures. This terminology is confusing and makes presumptions about the biochemical and morphological characteristics of *B. burgdorferi* RBs, such as a lack of cell wall (CWD, spheroplasts and protoplasts), or that these forms are encysted with a capsulated outer membrane (cysts). However, the cell envelope components and morphology of *B. burgdorferi* RBs have not been clearly studied before.

Although RBs of *B. burgdorferi* have been observed from limited *in vivo* clinical samples (Aberer *et al.*, 1996; Hulínská *et al.*, 1994; Mattman, 2001; Miklossy *et al.*, 2008), the role of pleomorphism in pathogenesis of Lyme disease and other diseases has been hugely debatable and recently criticized (Lantos *et al.*, 2014; Onwuamaegbu *et al.*, 2005; Schnell *et al.*, 2014). However, there is more and more plausible evidence that pleomorphism in general may help the bacteria to evade the immune system or decrease antibiotic susceptibility, as well as change its pathogenic mechanisms (Domingue & Woody, 1997; Justice *et al.*, 2008). This study provides a systematic in-depth compilation of *B. burgdorferi* pleomorphic variant characterization. The analysis of induction in different conditions, morphology, cell envelope architecture and metabolic activity as well as biochemical features of pleomorphic forms provides new insight into the morphological variants of *B. burgdorferi*. In order to fully understand the complex life cycle of *B. burgdorferi* and mechanisms of how pleomorphism is associated with diseases, it is crucial to understand what will induce different forms and what the basic features are that they convey.

## Methods

### 

#### Bacterial strain and growth conditions.

Infectious *B. burgdorferi* strain B31 was obtained from ATCC (ATCC 35210). Cultures were grown in Barbour–Stoenner–Kelly medium (BSK-II) without gelatin (Barbour, 1984), supplemented with 6 % rabbit serum at recommended temperature 37 °C (ATCC). The optimum growth temperature for *B. burgdorferi* B31 is reported to be 33 °C (Hubálek *et al.*, 1998); however, bacteria were cultured at physiologically relevant 37 °C. Low-passage number of bacteria, normally 1–2, was used in all experiments and utilized before cell density reached the late-exponential phase.

#### Imaging of live pleomorphic forms.

From bacterial cultures in the mid-exponential phase, 4 µl was mounted on a microscope slide to view spirochaetes, blebs and BFL aggregates. Spirochaetes were transformed to RBs by exposure to distilled H_2_O for 10 min. Samples were visualized using a Leica DM5500 fluorescence microscope with differential interference contrast microscopy (DIC) set-up and ×100 objective.

#### Induction of pleomorphic forms in different culturing environments.

Induction of pleomorphic forms was studied using complete BSK-II medium without 6 % rabbit serum, BSK-II medium supplements of with 10 % human serum (HS) or RPMI 1640 medium without the supplements of serum and BSA. To prevent complement-mediated cell lysis, the HS was heat-inactivated (see technical specifications; Sigma). Normal BSK-II medium with 6 % rabbit serum was used as a control. A total of 80×10^6^ cells were centrifuged at 5000 ***g*** for 10 min, resuspended in 4 ml appropriate medium and incubated at 37 °C for 4 days in order to reach the very-late-exponential phase of growth. Samples were prepared as triplicates. A moderately high initial density of bacteria was used to enable the counting with high magnification. After 2 and 4 days, 4 µl sample from each tube was prepared on the microscope slide and spirochaetes, blebs, RBs, BFL aggregates and cells with outer membrane damage were counted using a Leica DM5500 fluorescence microscope with phase-contrast (PH) set-up and ×100 objective.

#### Induction of pleomorphic forms of *B. burgdorferi* with distilled H_2_O.

Bacteria at mid-exponential phase were exposed to H_2_O for 10 min, 2 h, 4 h, 1 day and 4 days. Samples and controls were prepared and pleomorphic forms were counted at each exposure time as described above. To determine the mean diameter of RBs and blebs, 2 h H_2_O-induced RBs and spirochaetes with membrane blebs from control cultures in normal BSK-II medium were imaged with a Leica fluorescence microscope using PH and ×100 objective. The diameters of 100 RBs and blebs (approximately 33 per experiment) were measured from images using ImageJ software (NIH).

#### *B. burgdorferi* growth curve and BFL development.

Bacterial growth and development of BFL aggregates was examined by counting cell concentration and BFL colonies daily for 10 days from the stationary until the late-exponential phase. *B. burgdorferi* cultures with 2×10^4^ cells ml^−1^ were prepared as triplicates. Cells and biofilms were counted each day using a C-Chip DHC-N01 Disposable Haemocytometer (System Neubauer Improved; Digital Bio) and Leica fluorescence microscope with DIC and ×20 objective. Aggregates of more than ten cells were counted as BFL.

#### Reversion of RB forms to spirochaetes.

RBs were induced by H_2_O as described above and 60×10^6^ treated cells were centrifuged at 5000 ***g*** for 10 min, resuspended to a final concentration of 10×10^6^ cells ml^−1^ and incubated at 37 °C. Reversion cultures were viewed regularly every 2–4 days with DIC or PH microscopy to observe the transformation of RBs to normal spirochaetes. When there were signs of reversion, corresponding to a small amount of single motile spirochaetes, cultures were viewed more often until the growth reached exponential phase and concentration of approximately 40×10^6^ ml^−1^. Cultures that showed no reversion after 21 days were kept in the incubator for up to 3 months and were regularly checked for growth. In parallel, to address if bystander spirochaetes from H_2_O- induced RB cultures were able to replicate and interfere in the reversion experiments, 30×10^6^ treated cells were filtered using a Filtropur S 0.45 µm filter to remove RBs. The filtered suspension with possible bystander spirochaetes was centrifuged at 5000 ***g*** for 10 min, resuspended to 3 ml in BSK-II medium and incubated at 37 °C. The growth and morphology were observed as described above.

#### ATP determination assay.

ATP activity of parental spirochaetes, and 10 min and 2 h H_2_O-induced RBs were analysed using an ATP determination kit (Molecular Probes). To determine the increase in ATP metabolism during reversion, ATP activity of RBs reintroduced to normal medium with initial concentrations of 10×10^6^ ml^−1^ for 1 h, 24 h and even up to early- and mid-exponential phase of growth was assayed. The growth in reversion cultures was defined to be in early- and mid-exponential phase when the spirochaete counts were approximately 10–20×10^6^ and 40–50×10^6^, respectively. Spirochaetes treated with 100 µg doxycycline ml^−1^ for 24 h and methanol-killed spirochaetes were used as negative controls. The luminescence of all samples was measured in white opaque 96-well plates with 1×10^7^ cells per well using a Victor X Multilabel Plate Reader. The ATP concentrations were determined from a standard curve prepared with known ATP concentration standards provided by the kit.

#### Modelling of RB transformation.

RB formation was animated and rendered with Blender 2.69 (http://www.blender.org). The expanding outer membrane was modelled with a transparent sphere and made to grow in every step. The spirochaete outer membrane and protoplasmic cylinder were modelled with 3D Bézier curves. The shape of the internal Bézier curve was set manually for each time point, after which it was duplicated, cut on the edge of the sphere and enlarged to form an external spirochaete tail. The protoplasmic cylinder was set to green and opaque while outer membrane was set as transparent.

#### Morphological analysis with transmission electron microscopy (TEM).

The morphologies of spirochaetes, blebs and 2 h H_2_O and 4 days HS RBs, as well as the localization of the flagella, were studied using TEM. In addition, the different steps of RB formation, especially the folding of the protoplasmic cylinder, were examined and modelled. To obtain a high quantity of blebs, 25 ml of 2 h H_2_O RBs with a concentration of 40×10^6^ ml^−1^ were introduced to 25 ml normal culture medium for 1 h to induce RB unfolding to blebs. In addition, spirochaetes treated with 100 µg doxycycline ml^−1^ for 24 h were used as a control to demonstrate the damage at the outer membrane. All samples were prepared as duplicates with 1×10^9^ cells using a recently published protocol (Huttunen *et al.*, 2014), embedded in embedding resin medium, cut and finally visualized with a JEOL JEM1400 transmission electron microscope. The swollen RBs were counted in H_2_O and HS RB images to compare the amounts in these treatments. Protoplasmic cylinders with a diameter of approximately 200 nm were considered normal size. Where the diameter was >200 nm, they were counted as swollen. The total numbers of measured H_2_O and HS RBs were 171 and 212, respectively.

#### Immunolabelling of flagella.

Approximately 20×10^6^ spirochaetes, 2 h H_2_O RBs, and 4 days HS RBs were centrifuged at 9300 ***g*** for 5 min, fixed with ice-cold methanol for 20 min at −20 °C and washed with PBS. Cells were immunolabelled using a previously described protocol (Thammasri *et al.*, 2013). Here, primary anti-flagellin p41 antibody (Acris) (1 : 50) and secondary goat anti-mouse IgG conjugated with Alexa 488 (1 : 200) were used. Cells were mounted with Prolong Gold antifade reagent with DAPI (Molecular Probes) and visualized with an Olympus microscope IX81 with a FluoView-1000 confocal set-up with ×60 objective using 488 nm laser and DIC.

#### Composition analysis of pleomorphic forms with fluorescence microscopy.

Bacterial cultures (100 µl) were stained with 50 µg propidium iodide (PI) ml^−1^, or 10 µg wheatgerm agglutinin (WGA) ml^−1^ conjugated with Alexa 488 for 1 h at room temperature, or 50 µg boron-dipyrromethene (BODIPY 493/503) ml^−1^ for 1 h at 37 °C (followed by a wash with medium), or 1 % (w/v) acid fuchsin for 1 h at room temperature to indicate DNA, polysaccharides, lipids and collagen, respectively. Live cells with PI were imaged immediately. To address the specificity of the stains in live cells, cells were fixed with ice-cold methanol for 20 min and labelled with similar dyes for 10 min, washed with PBS and mounted with Prolong Gold with DAPI. BFL aggregates from late phase bacterial cultures were purified with MACS 30 µm pre-separation filters (Miltenyi Biotec) and washed with BSK-II medium to remove individual spirochaetes. Samples were visualized using an Olympus confocal IX81 microscope, ×60 objective, 488 or 546 nm laser and DIC. The Supplementary Movie S2, available in the online Supplementary Material, was acquired using a Nikon A1R confocal microscope with resonant scanning, ×60 objective and 561 nm laser.

#### Microscopy data analysis.

All microscopy data were processed and analysed using open source ImageJ software. The brightness and contrast settings of images were adjusted and applied to all parts of the image. The noise from green and red fluorescent images was suppressed using Gaussian blur filter with sigma (radius) 1–2. If needed, the uneven illumination was corrected in the DIC images using pseudocorrection.

## Results

### Various *in vitro* culturing environments can induce pleomorphism of *B. burgdorferi*

**Fig. 1.**
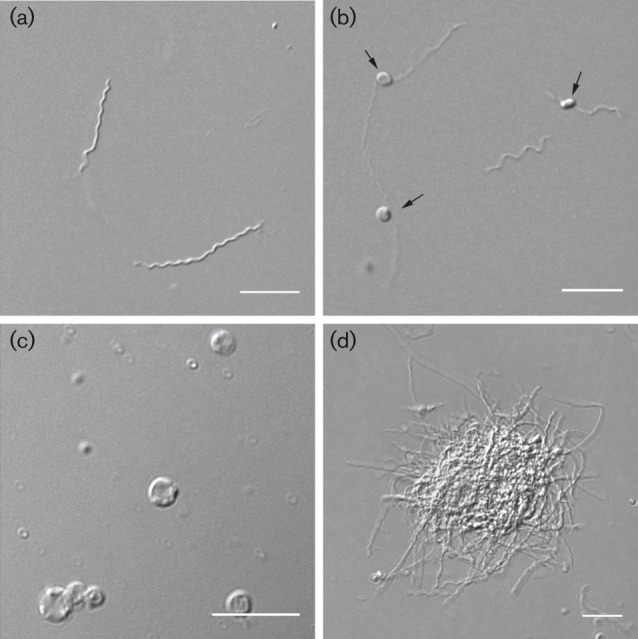
Typical pleomorphic forms of *B. burgdorferi* B31. Live cell DIC images of *B. burgdorferi* cultures representing (a) spirochaetes, (b) blebs on spirochaetes (black arrows), (c) 10 min H_2_O-induced RBs and (d) BFL aggregates. Bars, 10 µm.

**Fig. 2.**
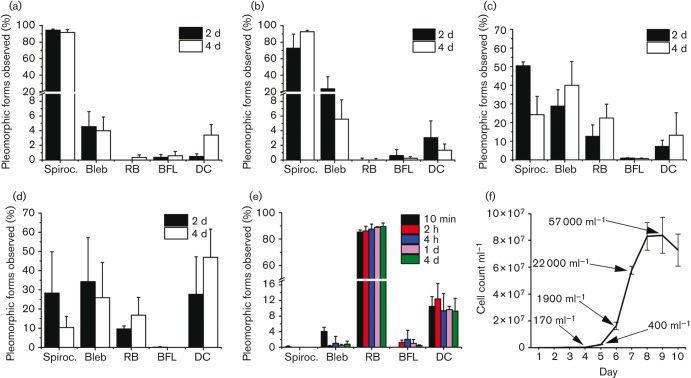
Unfavourable culturing conditions induced pleomorphic forms. The percentages of observed spirochaetes, blebs, RBs, BFL aggregates and cells with damaged outer membrane (DC) in (a) normal BSK-II, (b) serum-free, (c) HS, (d) mammalian culture medium and (e) distilled H_2_O culture conditions. Cells were viewed with PH microscopy using a ×100 objective and different forms were counted from each sample at 2 days and 4 days. The H_2_O RBs were counted at time points 10 min, 2 h, 4 h, 1 day and 4 days. Collective observations are provided from three independent experiments. In total, approximately 800 cells per treatment were counted. (f) Growth curve of 80 000 cells in a total volume of 4 ml was recorded. Arrows depict development of BFL colonies at days 4, 5, 6, 7 and 9 with colony count ml^−1^. Error bars illustrate sd from three experiments.

In this study, the induction of different pleomorphic forms in various culturing environments including HS was extensively examined. In addition, cells with outer membrane damage were quantified; however, these were not defined as pleomorphic. Here, we compiled the descriptions of different morphological variants based on our findings at a physiologically relevant culturing temperature of 37 °C (Table 1). It is notable that the mean size of RBs (2.8 µm) was greater when compared with the blebs (1.3 µm) on spirochaetes (Table 1). When in physiologically relevant *in vitro* culturing environment, *B. burgdorferi* is most commonly seen as a spirochaete (Fig. 1a), but other forms such as membrane blebs (Fig. 1b) and BFL aggregates (Fig. 1d) are also present in low levels (Fig. 2). Fig. 1(c) displays the spirochaetes converted to the smaller RBs when exposed to H_2_O for 10 min; however, these forms also exist in small numbers in normal culturing conditions (Fig. 2a).

**Table 1.** Description of different pleomorphic forms of *B. burgdorferi*

Form	Description of morphology	Size
Spirochaete	Long, corkscrew-shaped	Mean length, 20 µm
Bleb	Spirochaete with membrane bleb	Diameter of bleb, 1.3±0.43 µm*
RB	Spherical	Diameter, 2.8±0.46 µm*
BFL	Colony of mostly spirochaetes; however, blebs and RBs are commonly present. Contains EPS	Consists of more than 10 spirochaetes/blebs/RBs

* Mean±SD

In the standard culturing environment, BSK-II medium at 37 °C, the mean number of different pleomorphic forms remained the same during the whole culturing period from early-exponential phase until the late phase of growth up to day 4 (Fig. 2a). At 4 days, 92 % of the bacteria were parental spirochaetes, 4 % were blebs and 0.6 % were BFL aggregates. After 2 days RBs were not observed; however, after 4 days, 0.4 % of the bacteria were observed in this form. There is a basal level of damaged cells in cultures that increases with time. Here, the damaged cells in control cultures increased from 0.5 % to 3.4 % from day 2 to day 4. When bacteria were exposed to BSK-II medium without rabbit serum, there was little effect on bacteria when compared to controls (Fig. 2b). At day 2, bacteria had a stress reaction seen as an elevated number of blebs (24 %), but after 4 days the level was normalized back to 6 %. After 4 days there was exactly the same amount of spirochaetes (93 %) as in controls, and only 1.3 % of the cells were damaged. In addition, bacteria in BSK-II medium without rabbit serum replicated normally and maintained motility.

To simulate the physiological conditions in humans, *B. burgdorferi* was grown in BSK-II medium without supplement of BSA or rabbit serum, but instead with 10 % HS. Interestingly, the amount of spirochaetes decreased from 93 % to 24 % in 4 days, while blebs and RBs as well as the quantity of damaged cells increased to 40 %, 22 % and 13 %, respectively (Fig. 2c). The level of cell damage remained the same throughout the experiment. In addition, bacteria were exposed to the mammalian cell culture medium RPMI 1640 without supplement of FBS or antibiotics. RPMI medium clearly induced RBs and blebs, but also caused severe cell damage (Fig. 2d). After 4 days, only 10 % of the cells were spirochaetes. The level of RBs increased to 17 % during the 4 day experiment. Furthermore, the amount of blebs decreased from day 2 (34 %) to day 4 (26 %), whereas the amount of damaged cells increased from 28 % to as much as 47 %.

In order to study pleomorphic forms *in vitro*, it is important to develop methods to induce them easily in high quantities. Here, we followed a previously published method (Brorson & Brorson, 1998) with modifications to induce RBs with distilled H_2_O. The incubation times were shorter (10 min, 2 h, 4 h and 1 day) compared to the other culturing experiments, because RB formation in H_2_O was very rapid. The longer 4 day time point was included to examine the long-term effects in H_2_O. After 10 min 85 % of the cells were perceived as RBs (Fig. 2e). Only 0.1 % of the cells were seen as parental spirochaetes and 4 % as blebs. As expected, H_2_O caused cell damage, observed in 10 % of the cells throughout the experiment. From 2 h to 4 days of incubation, normal spirochaetes did not exist in the cultures. All the spirochaetes observed had damaged outer cell membranes, and blebs decreased to 1 % or less.

Suspended BFL aggregates were consistently present in all culture conditions, although at low levels of 2 % or less (Fig. 2a–c, e), except at day 4 in RPMI medium when aggregates were not observed (Fig. 2d). BFL colonies were detected early, even before cells reached the exponential phase of growth (Fig. 2f). Even at day 4 from the initial growth, when cell density was 3.7×10^6^ ml^−1^, the first aggregates were measured as having a concentration of 170 aggregates ml^−1^ (Fig. 2f). Along with the proliferation of the cells, the amount of biofilm also increased to 57 000 ml^−1^ in late-exponential phase at 9 days.

### RBs have the ability to become viable spirochaetes

**Fig. 3.**
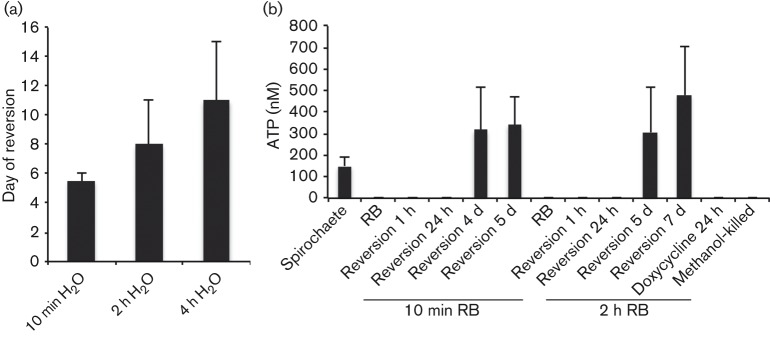
RBs do not have ATP activity but have the ability to revert to viable spirochaetes. (a) Mean reversion times of 10 min, 2 h and 4 h H_2_O RBs. RBs from each time point were reintroduced to a normal BSK-II medium and incubated at 37 °C until the spirochaete density reached 40×10^6^ ml^−1^. Error bars indicate sd from three different experiments. (b) ATP concentrations of spirochaetes, 10 min and 2 h H_2_O RBs, RBs reverted in BSK-II medium for 1 h, 24 h, as well as until early- and mid-exponential phase of growth (10–20×10^6^ ml^−1^ and 50×10^6^ ml^−1^, respectively). RBs exposed to H_2_O for 10 min reached the early growth phase in approximately 5 days whereas 2 h RBs achieved it at day 6. Mid-exponential phase growth was reached in approximately 6 days with 10 min RBs and in 8 days with 2 h RBs. Cells treated with 100 µg doxycycline ml^−1^ for 24 h and methanol-fixed cells were used as negative controls. Error bars indicate sd from three experiments.

To test viability, and to see if RBs were able to revert to their parent vegetative spirochaetes, RBs induced with H_2_O were transferred to normal culturing conditions. The mean reversion time was determined when the newly formed spirochaetes were in mid-exponential phase of growth with a cell density of 40×10^6^ ml^−1^. H_2_O-induced 10 min and 2 h RBs reverted to motile spirochaetes each time, and reached a density of 40×10^6^ ml^−1^ at 6 and 8 days, respectively (Fig. 3a). The 4 h RBs reverted in half of the cases, and the mean reversion time was 11 days. After 1 day or 4 days exposure to H_2_O, RBs did not revert even after 3 months of culturing. To confirm that bystander spirochaetes are not replicating and interfering with the reversion assay, the growth of filtered spirochaetes was monitored in parallel. Eventually, bystander spirochaete cultures did not show growth (data not shown).

### RBs display lower metabolic activity

ATP determination experiments indicated that 10 min and 2 h RBs do not have ATP production (Fig. 3b), while control spirochaetes in mid- or late-exponential phase of growth had a mean ATP concentration of 149 nM. Furthermore, when RBs were placed in normal culture medium to revert to spirochaete form, ATP activity was not detected after 1 h or 24 h. However, RBs were able to revert and become metabolically active spirochaetes. At approximately 5 days of culturing, 10 min RBs reached the early-exponential phase (10–20×10^6^ cells ml^−1^) with an ATP concentration of 317 nM (Fig. 3b). When cells were in mid-exponential phase (40–50×10^6^ cells ml^−1^) at approximately day 6, ATP production was almost the same, 343 nM. The 2 h RBs achieved the early-exponential phase at approximately day 6 and mid-exponential phase at day 8, demonstrating ATP production of 306 and 480 nM, respectively (Fig. 3b).

### Model for RB formation

**Fig. 4.**
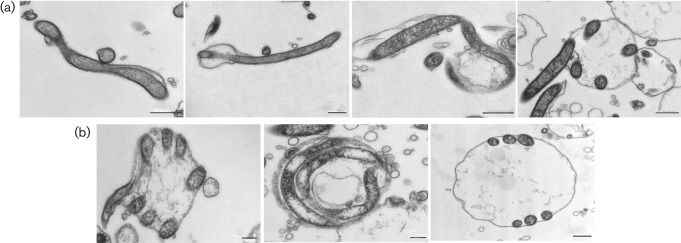
RB formation evolved through the expansion of the outer membrane and folding of the protoplasmic cylinder. (a) Stepwise demonstration of RB development using TEM images of H_2_O and HS RBs. Presented from left to right: parental spirochaete; spirochaete with initial membrane expansion; bleb where folding of the protoplasmic cylinder inside the outer membrane is initiated; bleb transitioning to the RB formation with folded protoplasmic cylinder under the expanded outer membrane. Bars, 500 nm. (b) Cross-sections of completed RBs and the organization of folded protoplasmic cylinder are visualized with TEM micrographs. RB is displayed from the side, from the front and from the top, respectively, from left to right. Bars, 200 nm, 200 nm and 500 nm, respectively.

The development of *B. burgdorferi* RBs proceeded in steps that are presented on TEM illustrations (Fig. 4a). *B. burgdorferi* has an elastic outer envelope that expands and allows folding of the protoplasmic cylinder within the cell. This leads to the transformation of spirochaetal corkscrew morphology to a spherical shape. The completed RB with folded protoplasmic cylinder is presented in three different cross sections in Fig. 4(b). In addition, an animation (Movie S1) that is based on observations from TEM and DIC/PH imaging is provided to demonstrate this folding.

### Pleomorphic forms have a cell wall

**Fig. 5.**
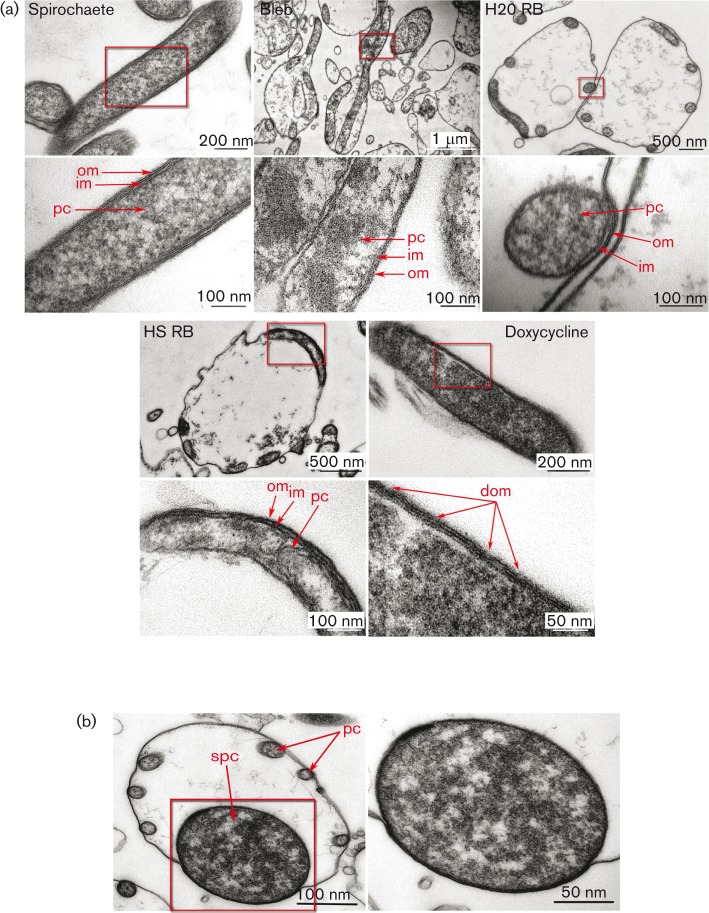
Pleomorphic forms of *B. burgdorferi* are not CWD. (a) TEM micrographs of Epon-embedded spirochaetes, blebs, 2 h H_2_O RBs, 4 days HS RBs and cells with outer membrane damage induced by treatment with 100 µg doxycycline ml^−1^ for 24 h. Blebs were prepared by induction of 2 h H_2_O RBs and were then reintroduced to normal culture medium and incubated for 1 h at 37 °C. The upper panels represent the overall view of the whole cell, and expanded insets in the lower panels indicate the zoomed morphology of the outer envelope. The outer membrane (om), inner membrane (im) and protoplasmic cylinder (pc) are easily discernible in zoomed images. The lower panel of the doxycycline-treated spirochaete illustrates the damaged outer membrane (dom). (b) TEM images of swollen 4 days HS RB with swollen protoplasmic cylinder. Left panel illustrates the RB with normal size protoplasmic cylinders (pc) and one swollen protoplasmic cylinder (spc). Right panel displays the zoomed view of the swollen protoplasmic cylinder.

To further investigate the morphology and the cell wall characteristics of different *B. burgdorferi* forms, spirochaetes, RBs and blebs were analysed using TEM. Doxycycline-treated cells were used as a control for outer cell envelope damage. Fig. 5 presents morphologies of spirochaete, bleb, H_2_O RB, HS RB and doxycycline-treated spirochaete, respectively (top panels). Insets depict magnified areas (lower panels). Arrows highlight the outer and inner membranes as well as the protoplasmic cylinder. Blebs were revealed to be an intermediate stage between the spirochaete and the RB, with an expanded outer envelope usually on the other end or on the lateral part of the spirochaete with a partly folded protoplasmic cylinder inside (Figs 4a and 5a). TEM images clearly indicated that both H_2_O and HS RBs have intact double outer and inner membranes around the protoplasmic cylinder, similar to the spirochaete (Fig. 5a). Doxycycline-treated cells displayed more damage seen as small holes on the outer membrane when compared to the other forms (Fig. 5a). Interestingly, TEM also unveiled that 28 % of the HS RBs displayed swollen protoplasmic cylinders, while only 4 % of the H_2_O RBs were swollen (Fig. 5b).

### Flagella are present in RBs

**Fig. 6.**
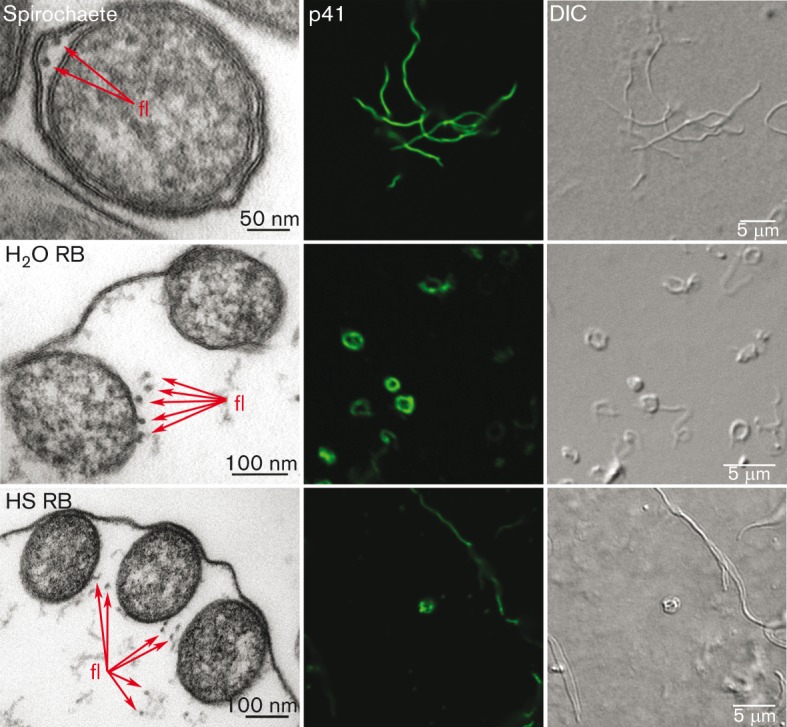
Flagella are present in RBs. TEM and confocal micrographs of flagella localization in spirochaetes, 2 h H_2_O RBs and 4 days HS RBs. TEM images (left panels) demonstrate the cross-section of the cell, where flagella (fl) are indicated by arrows. Middle panels display confocal images of cells immunolabelled with p41 flagellin protein antibody and Alexa 488 (green). Right panels illustrate the DIC image.

The localization of flagella in RBs was examined using TEM and fluorescence microscopy. In spirochaetes, the flagella are localized in the periplasmic space between the outer and inner membrane, as seen in Fig. 6. Fluorescence images of cells immunolabelled with p41 antibody indicated that the flagella extend through the whole spirochaete. In RBs, the expanded periplasmic space is presented quite differently compared to the spirochaete (Figs 4a, b and 5a); nonetheless, the flagella are visualized inside the RBs (Fig. 6).

### *B. burgdorferi* pleomorphic forms have atypical cell wall characteristics

**Fig. 7.**
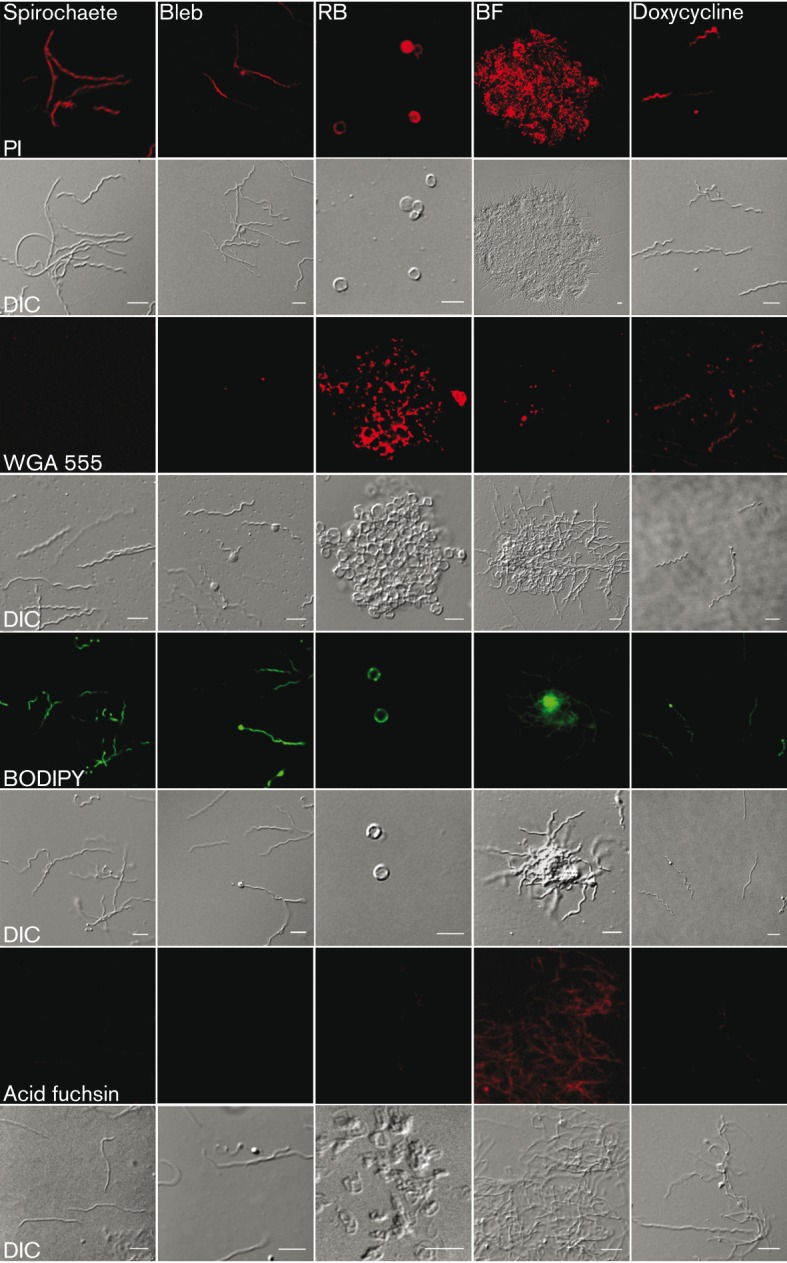
Composition analysis of *B. burgdorferi* indicates the distinction between the different pleomorphic forms and presents atypical cell wall characteristics. Live spirochaetes, blebs observed in normal culture conditions, 2 h H_2_O RBs and BFL aggregates in suspension as well as 24 h doxycycline-treated damaged cells were stained with PI, WGA conjugated with Alexa 555 (WGA 555) and BODIPY to indicate DNA, polysaccharides and lipids. Acid fuchsin was used for methanol-fixed cells to stain collagen. Upper panel visualizes the cells imaged with confocal laser scanning microscopy. Lower panel represents the morphology of the cells with DIC. Bars, 5 µm.

Live pleomorphic variants of *B. burgdorferi* were stained with several dyes and imaged with laser scanning confocal microscopy to address different cell envelope components (Fig. 7). As a control, the same dyes were used on doxycycline-treated (Fig. 7) and methanol-fixed cells (Fig. S1). This demonstrates the difference in the capability of the dyes to penetrate intact and damaged cell membranes. PI is a DNA stain that is thought to be unable to permeate intact cell membranes. However, here we demonstrated that PI stained live motile spirochaetes (Fig. 7, Movie S2), demonstrating the unique, atypical Gram-negative outer envelope of *B. burgdorferi*. In addition PI penetrated all different live forms (blebs, RBs, BFL aggregates) and doxycycline-treated cells. WGA 555 was used to label *N*-acetylglucosamine polysaccharides (GluNAc), the structural component of the bacterial peptidoglycan cell wall. Intriguingly, WGA stained the cell wall of the RBs very specifically. Spirochaetes, blebs and BFL aggregates did not have labelled GluNAc while doxycycline-treated and methanol-fixed cells displayed staining (Figs 7 and S1), presumably because of the leakage through the damaged outer membrane. As expected, the lipid-binding BODIPY stained all the *B. burgdorferi* variants. Because acid fuchsin is suitable only on fixed cells, live cell imaging could not be performed with this particular dye. Nevertheless, we used fixed cells to indicate staining of collagen on the bacteria. As a result, only BFL colonies were stained, indicating that the suspension biofilms observed in normal cultures have proteins, especially collagen, on their extracellular polymeric substance (EPS) matrix (Fig. 7). This provides more evidence that these cultured BFL aggregates of *B. burgdorferi* share characteristics with surface-bound biofilms (Flemming & Wingender, 2010). Furthermore, fixed cells with permeabilized cell envelopes clearly had a more robust internal staining pattern, indicating that all pleomorphic variants have an intact cell envelope.

## Discussion

In addition to typical spirochaetes, *B. burgdorferi* has pleomorphic forms present in normal culturing conditions although in low quantities (Figs 1 and 2a), raising the question of whether these forms are part of the *B. burgdorferi* normal life cycle and how that may affect pathogenesis of the disease. Blebs, similar to those seen within this study (Figs 1b and 2a), have been detected earlier during standard *in vivo* and *in vitro* culturing at 34 °C (Garon *et al.*, 1989; Miklossy *et al.*, 2008; Radolf *et al.*, 1994). It is known that different disturbances, such as antibiotics, ageing and complement factors (Barbour & Hayes, 1986), can cause bleb development in *B. burgdorferi*, and this supports our findings that the formation of blebs increases under environmental stress. Blebs contain tightly packed DNA and it is suggested that they may be involved in transfer of genetic material and have certain protectoral functions (Garon *et al.*, 1989). Their role in the initiation of autoimmune disease processes is also proposed (Whitmire & Garon, 1993). Nevertheless, the function of these blebs is still relatively unknown.

Interestingly, transformation of spirochaetes to RBs increased remarkably when growth conditions changed to medium with HS, nutrient-poor mammalian RPMI culture medium or H_2_O (Fig. 2). The effect of HS on the morphology of *B. burgdorferi* has not been examined before, and growth in this environmental circumstance remarkably increased the quantity of blebs and RBs without causing extensive cellular damage (Fig. 2c). Growth in hypotonic conditions with distilled H_2_O at 37 °C (Figs 1c and 2e) induced similar morphologies, as previously described at 30–35 °C (Brorson & Brorson, 1998; Murgia & Cinco, 2004). The treatment with HS and distilled H_2_O induced morphologically similar RBs, indicating that the transformation does not occur only because of the osmotic stress. Rabbit serum normally supplementing the *B. burgdorferi* culture medium has the same osmolarity as the HS. Most probably, the complement system or antibodies in the serum are responsible for the changes in the morphology, which is clinically interesting and worthy of further studies. An antibody–complement system in HS has been found to be lethal for some L-forms (McGee *et al.*, 1972). Here, the bacteriolytic effect of the serum components was also seen in one-third of the RBs (Fig. 5b) as protoplasmic cylinder swelling.

RPMI medium induced RBs and blebs; however, it also triggered severe cell damage (Fig. 2d). This is distinctly different from the 95 % RBs that were reported after 2 days culture in similar media (Alban *et al.*, 2000). With respect to culturing in rabbit serum-free BSK-II medium, RB formation did not occur in growth at 37 °C up to 4 days (Fig. 2b). Others have documented RBs in cultures at 33–34 °C after 7–10 days (Al-Robaiy *et al.*, 2010) or six weeks (Brorson & Brorson, 1997). The dissimilarity in these observations may be due to the different growth temperatures, where 37 °C actually provides culturing conditions that better suit the maintenance of the bacterial growth. Long-term cultures of up to six weeks in rabbit serum-free medium may have indeed induced RBs, although this was not mentioned by other authors. In addition, these studies used relatively low magnification (approximately ×200) for counting the different morphologies, but here we used ×1000 magnification to enhance the actual distinction between different pleomorphic forms and damaged cells.

*In vitro* growth of *B. burgdorferi* biofilm colonies both on surfaces and in suspension at 33 °C or at 35 °C has been displayed in several studies (Barbour, 1984; Sapi *et al.*, 2012; Srivastava & de Silva, 2009). High cell density promotes BFL formation of *B. burgdorferi in vitro* (Srivastava & de Silva, 2009). Here, there is evidence that BFL colonies are established even at early-exponential phase of growth (Fig. 2f), suggesting that high density is not the only factor enhancing biofilm formation in suspension. Suspended *B. burgdorferi* biofilms share common characteristics with surface-attached biofilms such as alginate polysaccharide and extracellular DNA on the matrix of EPS (Sapi *et al.*, 2012). We demonstrated here that collagen is present on EPS of *B. burgdorferi* BFL colonies in suspension (Fig. 7), supporting the previous findings that suspended biofilms are biofilms, not just cell aggregates. Furthermore, DIC images (Figs. 1d and 7) provide visual evidence that these biofilms have cellular architecture similar to surface biofilms (Serra *et al.*, 2013). There is a lack of literature that correlates *B. burgdorferi* biofilms with clinical consequences; however, it is speculated (Barbour, 1984) that bacterial aggregation may enhance the binding of bacteria to host tissue with the avoidance of phagocytosis. The presence of collagen-like protein in EPS may indeed encourage *B. burgdorferi* suspension biofilm binding to host tissue.

It has been suggested that transformation of *B. burgdorferi* from spirochaetes to RBs may enhance survival in inconvenient environmental conditions (Murgia & Cinco, 2004) and evasion from the immune system (Al-Robaiy *et al.*, 2010; Brorson & Brorson, 1998; Lawrence *et al.*, 1995). Remarkably, we found that RBs have lower metabolic activity (Fig. 3b); however, they have the ability to revert to spirochaete form and regain their ATP activity. Low metabolic rate may indeed enhance the survival of bacteria during antibiotic treatment. It remains unknown whether the RBs can utilize something other than the ATP metabolic pathway. Within this study, when H_2_O-induced RBs were grown in normal culturing medium at 37 °C, they were able to revert to viable spirochaetes only if the exposure to H_2_O was not more than 4 h. Previous studies (Al-Robaiy *et al.*, 2010; Brorson & Brorson, 1998; Murgia & Cinco, 2004) have presented the reversion of 1 day, 4 days, 7 days or 5 weeks in 30–34 °C H_2_O-exposed RBs, but we did not see reversion of 1 day and 4 days H_2_O-exposed RBs. Again, the difference in these observations could be due to the different culturing temperatures. However, it seemed that *B. burgdorferi* RBs could tolerate short exposure to harsh environment but not long-term exposures.

We provide a step-by-step model, which is in harmony with the findings of others (Al-Robaiy *et al.*, 2010; Murgia & Cinco, 2004), that the outer membrane of *B. burgdorferi* is flexible, allowing it to expand during RB transformation when the protoplasmic cylinder is folding within the envelope (Figs 4 and 5a, Movie S1). Furthermore, the flagella, located in the periplasmic space in spirochaetes (Barbour & Hayes, 1986; Zhao *et al.*, 2013), were present in RBs, indicating that these forms can maintain the motility and skeletal components and then recruit them again during reversion to spirochaetes (Fig. 6). It is suggested that the export system is anchored between the outer and inner membrane (Radolf *et al.*, 2012). However, our model indicates that the protoplasmic cylinder is flexible and not tightly anchored to the outer and inner membrane, allowing it to fold within the confinements of the RB outer envelope (Movie S1). *B. burgdorferi* is known to utilize the RND transporter system, an efflux pump for toxic compounds, situated on the outer and inner membranes in Gram-negative bacteria. They are thought to be associated with antibiotic resistance in *B. burgdorferi* (Bunikis *et al.*, 2008; Li & Nikaido, 2004). The adaptor protein in the RND transporter efflux pump in *B. burgdorferi* lacks a hairpin domain, which results in a smaller interaction with the outer and inner membrane components, leading to a less stable assembly of the pump (Bunikis *et al.*, 2008). This supports our observations about the flexibility of the *B. burgdorferi* outer membrane. The loose assembly of the efflux pump components could allow pump function even during RB formation.

*B. burgdorferi* is known to have an atypical Gram-negative cell membrane (Barbour & Hayes, 1986). However, this phenomenon of the unique cell wall properties and the consequences of it have not been widely discussed. Here we demonstrated the unique nature of the *B. burgdorferi* cell envelope by showing that exclusion stains, such as PI tested here, are able to enter living cells (Movie S2). PI is commonly used to identify dead cells or cells with compromised membranes, and caution is needed when interpreting PI staining results, especially to indicate cell death of *B. burgdorferi*. Intriguingly, RBs were seen to have a very specific binding of WGA to GluNac, indicating the differences in polysaccharide composition from other forms. This is in harmony with a previous study (Hulínská *et al.*, 1994), where RBs, but not spirochaetes, were found to be positive for WGA in *B. burgdorferi*-infected Langerhans cells. The advantage of having adaptable *in vivo* conformations could allow the bacteria to evade the immune system, leading to a persistent stage while still having efflux capabilities seen in the parent form. The peptidoglycan layer of *B. burgdorferi* is thought to be located close to the inner membrane (Radolf *et al.*, 2012); however, others (Kudryashev *et al.*, 2009) have presented that the layer is actually located in the periplasmic space near the outer membrane. Elasticity of the outer membrane and reorganization of the membrane components during RB formation could explain why GluNAc is being exposed.

Here we confirmed for the first time that RBs actually have an intact cell envelope with a peptidoglycan layer (Figs 5 and 7), indicating that they do not fulfil clearly the definitions of spheroplasts, CWD or encysted forms although there are some modifications in the cell envelope and cell wall architecture. Furthermore, the intact cell envelope of RBs (Fig. 5), similar to the spirochaete, provides evidence for the previous suggestion (Alban *et al.*, 2000) that RBs are not degrading cells. To avoid confusing terminology, we suggest that *B. burgdorferi* spherical shapes are termed ‘round bodies’ to describe these forms better.

Taken together, these results implied that pleomorphic forms of *B. burgdorferi* can be easily induced in different culturing environments with the presence of serum components, nutrient starvation or osmotic stress; however, low levels of the observed variants are present in normal culturing conditions at 37 °C. Our findings reassert the unique features of *B. burgdorferi* spirochaetes and their morphological variants: RBs have cell wll and flagella within the intact outer membranes. Furthermore, RBs and BFL colonies in suspension displayed specific staining properties when compared to other forms. Reorganization or modification of the cell envelope components that leads to exposure of the peptidoglycan layer in RBs could be exploited in diagnostics and recognition of RBs in tissue samples.
